# The level of consciousness and mental reactions of children after acute brain injury (interdisciplinary rehabilitation)

**DOI:** 10.1192/j.eurpsy.2024.1484

**Published:** 2024-08-27

**Authors:** D. Martyshevskaya, A. Zakrepina, Y. Sidneva

**Affiliations:** ^1^ Clinical and Research Institute of Emergency Pediatric Surgery and Trauma (CRIEPST); ^2^Federal state budgetary scientific institution “Institute of correctional pedagogy”/ ICP/; ^3^N.N.Burdenko National Medical Research Center of Neurosurgery, Moscow, Russian Federation

## Abstract

**Introduction:**

The process of recovery of mental reactions in children after acute traumatic brain injury is determined by complex methods with an interdisciplinary approach. Studies of emotional, communicative and behavioral reactions are based on an assessment by a psychiatrist and a teacher-defectologist.

**Objectives:**

to study mental reactions and identify predictors of positive recovery of consciousness after acute brain injury in children in early rehabilitation.

**Methods:**

psychiatric and pedagogical examinations; also - neuroimaging data and others.

**Results:**

Three groups of children were identified, depending on the different severity of emotional, communicative and behavioral indicators:

Group 1 (11%): The level of consciousness is minimal positive. Reactions: stable gaze fixation; emotional reaction to sound (smile) and the face of an adult; short-term tracking of the gaze of the object; the ability to touch an object and hold it; sits himself.

Group 2 (33%): The level of consciousness is minimal positive / negative, with an advantage of positive. Reactions: unstable gaze fixation; emotional reaction and involuntary movements to sound; reflex seizure of an object; sits with support.

Group 3 (56%): The level of consciousness is minimal negative. Reactions: no emotional reactions, low motor and sensorimotor activity.

**Conclusions:**

predictors of emotional-communicative and behavioral indicators of recovery of the level of consciousness were identified: sensory and motor, cognitive and socially-oriented (orienting reactions to the voice and face of an adult, tracking the gaze of an object, sensory and motor activity, etc.). These predictors are the basis for choosing a rehabilitation program with interdisciplinary support and a treatment strategy.

**Disclosure of Interest:**

None Declared

